# Genome Editing of the *SNAI1* Gene in Rhabdomyosarcoma: A Novel Model for Studies of Its Role

**DOI:** 10.3390/cells9051095

**Published:** 2020-04-28

**Authors:** Aleksandra Ulman, Klaudia Skrzypek, Paweł Konieczny, Claudio Mussolino, Toni Cathomen, Marcin Majka

**Affiliations:** 1 Department of Transplantation, Faculty of Medicine, Institute of Pediatrics, Jagiellonian University Medical College, 30-663 Cracow, Poland; 2 Institute for Transfusion Medicine and Gene Therapy, Medical Center – University of Freiburg, 79106 Freiburg, Germany; 3 Center for Chronic Immunodeficiency, Faculty of Medicine, University of Freiburg, 79106 Freiburg, Germany

**Keywords:** SNAI1 (SNAIL) transcription factor, rhabdomyosarcoma (RMS), genome editing, designer nucleases, CRISPR/Cas9, TALEN, shRNA

## Abstract

Genome editing (GE) tools and RNA interference technology enable the modulation of gene expression in cancer research. While GE mediated by clustered regularly interspaced short palindromic repeats (CRISPR)/Cas9 or transcription activator-like effector nucleases (TALEN) activity can be used to induce gene knockouts, shRNA interacts with the targeted transcript, resulting in gene knockdown. Here, we compare three different methods for *SNAI1* knockout or knockdown in rhabdomyosarcoma (RMS) cells. RMS is the most common sarcoma in children and its development has been previously associated with SNAI1 transcription factor activity. To investigate the role of SNAI1 in RMS development, we compared CRISPR/Cas9, TALEN, and shRNA tools to identify the most efficient tool for the modulation of SNAI1 expression with biological effects. Subsequently, the genome sequence, transcript levels, and protein expression of SNAI1 were evaluated. The modulation of SNAI1 using three different approaches affected the morphology of the cells and modulated the expression of myogenic factors and HDAC1. Our study revealed a similar effectiveness of the tested methods. Nevertheless, the low efficiency of the GE tools was a limiting factor in obtaining biallelic gene knockouts. To conclude, we established and characterized three different models of *SNAI1* knockout and knockdown that might be used in further studies investigating the role of SNAI1 in RMS.

## 1. Introduction

Since the discovery of DNA structure, genome and transcriptome modification techniques have been extensively developed. The most frequently used are RNA interference (RNAi) and genome editing (GE) with custom nucleases.

RNAi is based on an interaction between short RNA sequences, around 21–25 nucleotides, which regulate the expression through the degradation of mRNA, blocking of translation, or induction of epigenetic gene silencing [1]. The regulatory pathway is determined by the degree of complementarity between the short RNA and target mRNA. Perfectly matched sequences are degraded as a result of a dedicated RNA-induced silencing complex (RISC) activity [2]. Partially mismatched targets can be blocked or epigenetically silenced [3,4]. Short RNAs are generated from encoded short hairpin (sh)RNA precursors via enzymatic processing [5]. ShRNA could be incorporated into the genome with a viral vector to stably knockdown targeted mRNA [6] or with a plasmid [7] for transient expression modulation. Therefore, insertional mutagenesis, off-targets, and disturbance of the natural gene expression regulation machinery are the main obstacles when applying this technology [8].

Clustered regularly interspaced short palindromic repeats (CRISPR)/Cas9 together with transcription activator-like effector nucleases (TALEN) represent the main genome editing techniques. Their activity leads to DNA double-strand breaks (DSB) [9] that could be repaired via non-homologous end joining (NHEJ) or homology-directed repair (HDR). So-called “error prone” NHEJ is based on Ku protein activity and usually results in small deletions or insertions at the DSB. This, in turn, may lead to frameshift mutations and consequently to the gene knockout (KO) [10,11]. HDR requires a repair template with homologous arms to the cleavage site. When incorporated into the host genome, it may allow for the generation of precise gene knock-in or knockout [12]. The most frequently used CRISPR/Cas9 system II originates from *Streptococcus pyogenes* and is an analog of the bacteria’s adaptive immunity against invader nucleic acids. Similar to the RNAi system, the selectivity of the CRISPR/Cas9 is determined by the Watson–Crick base pairing of the guide RNA (gRNA) with a target DNA sequence. Endonuclease Cas9 is responsible for the generation of DSB in the genome. Prevention against self-digestion is guaranteed by the presence of a protospacer adjacent motif (PAM), which are specific trinucleotides near the gRNA recognition site [13,14,15]. TAL effectors from *Xanthomonas sp.* with their properties to regulate gene expression during plant pathogenesis were a precursor for developing the TALEN system [16]. TALEN are composed of highly conserved repeats, each made of 33–35 amino acids that bind to the DNA and an endonuclease domain, usually *FokI*, which is fused to it [17,18]. The specificity of the system is determined by the 12th and 13th amino acids in each repeat with the ability to recognize a dedicated nucleotide. The main obstacles in GE system applications are off-targets, which are unspecific DNA cleavages that can cause unwanted mutations in the genome. Nevertheless, custom nucleases are being applied in many studies, especially those investigating tumor biology [19,20], and they have entered clinical practice [21].

Rhabdomyosarcoma (RMS) is one of the most common soft tissue malignancies with a mesenchymal origin, occurring mostly in children and adolescents [22]. Defects during the myogenic differentiation process are associated with RMS development [23,24]. According to the histopathological analysis, a more aggressive form of cancer was distinguished, namely alveolar rhabdomyosarcoma (ARMS), which constitutes around 30% of all cases [25]. The poor prognosis of ARMS is associated with the PAX3/7-FOXO1 translocation and the increased expression of tyrosine kinase receptor MET, which is involved in the metastasis process [26,27,28]. In our previous work, the correlation between the upregulation of the SNAI1 (SNAIL) transcription factor expression in ARMS cell lines and patients’ tumors was shown [29]. Moreover, we demonstrated that SNAI1 plays a crucial role in ARMS differentiation by regulating the activity or expression of the myogenic regulatory factors (MRF) MYOD and MYF5, and the myogenic microRNAs miR-206 and miR-1. The downregulation of SNAI1 by shRNA results in the arrest of tumor growth *in vivo*, suggesting that SNAI1 is a crucial factor in ARMS growth [30].

SNAI1 is a member of the SNAIL zinc-finger transcription factor family. It is encoded by three exons, located on the 20th chromosome [31]. SNAI1 is well known for promoting the mesenchymal phenotype during the epithelial to mesenchymal transition by binding to the E-box motif in the E-cadherin promoter, which results in the repression of its expression and contributes to cancer invasiveness [32,33]. Recently, we have discovered that in mesenchymal tumors, such as RMS, SNAI1 plays a crucial role in myogenesis and cell cycle regulation [30].

In our current study, we applied and compared different tools for the modulation of SNAI1 expression in the ARMS cell line RH30 to develop a model for further research. CRISPR/Cas9 nucleases and TALEN were designed and optimized on HEK293T cells to assess their effectiveness at targeting the *SNAI1* gene. Next, the first and second *SNAI1* exons were simultaneously edited in RH30 cells using CRISPR/Cas9 and TALEN, respectively. We have also established an RH30 cell line with a stable downregulation of SNAI1 level after transduction with shRNA lentiviral vectors. Subsequently, we compared the *SNAI1* expression in three models at the mRNA and protein levels. We discovered that the modulation of the SNAI1 level regulated the expression of genes associated with myogenic differentiation.

## 2. Materials and Methods

### 2.1. Cell Culture

The ARMS RH30 cell line was kindly provided by Dr. PJ Houghton (Center for Childhood Cancer, Columbus, OH, USA). The cells were cultured in a high-glucose Dulbecco’s modified Eagle’s medium (DMEM; Lonza Group Ltd., Basel, Switzerland) supplemented with 10% fetal bovine serum (FBS; EURx, Gdansk, Poland) and 50 µg/mL gentamicin (Lonza) at 37 °C, 5% CO_2_, and 95% humidity. The cell lines were routinely tested for *Mycoplasma* contamination using a MycoAlert™ Mycoplasma Detection Kit (Lonza). Cell line authentication was performed using short tandem repeat (STR) profiling, as described previously [30].

HEK293T cells were cultured in DMEM (PAA Laboratories GmbH, Pasching, Austria) supplemented with 10% fetal calf serum (PAA Laboratories GmbH), 100 U/mL penicillin (PAA Laboratories GmbH), and 100 g/mL streptomycin (PAA Laboratories GmbH).

### 2.2. Design and Cleavage Activity of TALEN and CRISPR/Cas9 Nucleases Targeting the SNAI1 Gene

All TALENs were generated using standard cloning procedures, as described previously [34]. They targeted the following sequences of *SNAI1* gene 5′-3′:

TALENS Ex1 targeting *SNAI1* exon 1:

TALEN left: TCTTTCCTCGTCAGGAAGC

TALEN right: TGTAGTTAGGCTTCCGATT

TALENS Ex2B targeting *SNAI1* exon 2 

TALEN left: TTTACCTTCCAGCAGCCCT

TALEN right: TGGGATGGCTGCCAGCAGG

TALENS Ex2M targeting *SNAI1* exon 2 

TALEN left: TCCAGGAGAGTCCCAGGGT

TALEN right: TGTCCTCATCTGACAGGGA

CRIPSR/Cas9 plasmids targeting the *SNAI1* gene were generated using standard cloning procedures, as described previously [35]. gRNA represented the following sequences:

CRISPR exon 1 Ex1: GCTGTAGTTAGGCTTCCGATTGG

CRISPR exon 2 Ex2: GTGGGATGGCTGCCAGCAGGTG

HEK293T cells were transfected with plasmids encoding TALEN or CRISPR/Cas9 using polyethylenimine (PEI), as described previously [34]. To verify the TALEN’s expression in HEK293T cells, Western blot analysis was performed, as described previously [34]. TALEN or β-actin were detected with an anti-HA tag (1:2000; Novus Biologicals, Centennial, CO, USA) or anti-β-actin (1:2000; Cell Signaling, Leiden, The Netherlands) antibodies, respectively, and visualized with an HRP-conjugated anti-rabbit antibody (Dianova, Hamburg, Germany) and a West Pico Chemiluminescence substrate (Thermo Scientific, Waltham, MA, USA).

To amplify the sequences targeted by CRISPR and TALEN, PCR was performed using Phusion polymerase (Finnzymes, Espoo, Finland). Sequences of the used primers are presented below:

*SNAI1* exon 1:

For: 5′-CCGGAGTACTTAAGGGAGTTG-3’

Rev: 5′ -CTCGATCCTGGCTCAGG-3’

*SNAI1* exon 2:

For: 5′-CAGGAACCTGGTCTGTCC-3’

Rev: 5′-CTTTCGAGCCTGGAGATCC-3’

After the reaction, the PCR products were purified using a GeneMATRIX Basic DNA Purification Kit (EURx). A 10× NEBuffer 2 (New England Bio Labs, Rowley, MA, USA) was added to the total volume of the previously purified PCR products to the final concentration 1×. The prepared solution was then incubated for 5 min at 95 °C and slowly cooled down until it reached room temperature. Next, 200 ng of the prepared PCR products was diluted in NEBuffer 2 to produce the final volume of 12 μL and 4 U of T7 endonuclease (New England Bio Labs) was added to the mix. An analogous solution without the enzyme was also prepared to act as a negative control. The enzymatic reaction was carried out for 30 min in a 37 °C water bath. Products after the reaction were mixed with 6× Loading Buffer Yellow (EURx) and separated in 1.5% agarose gel (EURx) with 0.5 μg/mL ethidium bromide (Sigma-Aldrich, St. Louis, MO, USA). The fragmented PCR products were analyzed to determine the percentage of nuclease-specific cleavage products (fraction cleaved) using Image Lab 6.0.1 software (BioRad, Hercules, CA, USA). The estimated gene modification was calculated using the following formula: % gene modification = 100 × (1 – (1 − fraction cleaved)^1/2^), as described previously [36].

### 2.3. Bioinformatical Validation of Off-Targets

#### 2.3.1. CRISPR/Cas9

CRISPR/Cas9 gRNA1 and gRNA2 were analyzed according to the potential off-targets. We used the CCTop predictor, available at the following website https://crispr.cos.uni-heidelberg.de/ with particular settings: NGG where “N” indicates any nucleotide base, PAM (protospacer adjacent motif) type (*Streptococcus pyogenes*), targeted site length 20, total mismatches (MM) 4, core length 12, core MM 2, species human (*Homo sapiens* GRCh38/hg38) [37].

#### 2.3.2. TALEN

Potential TALEN pairs of off-targets were verified with PROGNOS (predicted report of genome-wide nuclease off-target sites) from the http://baolab.rice.edu/cgi-bin/prognos/prognos.cgi website. TALEN was set as a nuclease type; the sequence was entered as Top Strand, and the repeat variable diresidue (RVD) input format was numerical. Four was established as the maximum number of mismatches per half-site and other setting were default [38].

### 2.4. Transfection of RH30 Cells with Constructs Encoding TALEN and CRISPR/Cas9 Nucleases Targeting the SNAI1 Gene

The cells were transfected with Lipofectamine 2000 (Invitrogen, Carlsbad, CA, USA) according to the vendor’s protocol. Briefly, the day before transfection, the RH30 cells were seeded on a 24-well plate at a density of 70,000 cells per well. A total of 1 μg of plasmids coding CRISPR/Cas9 or TALEN systems were mixed with 1 μL of Lipofectamine 2000 and added to the cells. The gRNA plasmids to Cas9 plasmid ratio was 1:3. To each reaction, 200 ng of mCherry plasmid was added.

### 2.5. Transduction of the RH30 Cells with shRNA Lentiviral Vectors

The RH30 cells were transduced with shRNA lentiviral particles targeting *SNAI1* (a mixture of three different sequences) and control lentiviral particles at an MOI of 2.5 (Santa Cruz Biotechnology, Santa Cruz, CA, USA; sc-38398-V and sc-108080), and stable cell lines were developed as described previously [30].

### 2.6. Analysis of RH30 Clones

Forty-eight hours after transfection, the RH30 cells were harvested for gDNA analysis and single-cell clone isolation. Clones were seeded on a 96-well plate at a density of 0.5 cells per well.

gDNA was extracted from each clone with a Cell Culture DNA Purification Kit (EURx) according to the vendor’s protocol. The concentration of gDNA was measured with Nanodrop One (Thermo Scientific) or Quawell Q5000 (Quawell Technology, Inc., San Jose, CA, USA). The deletion verification was performed with a PCR reaction with Color OptiTaq PCR Master Mix (2×) (EURx) using the following primers:

For: 5’-TCGGAAGCCTAACTACAGCGA-3’

Rev: 5’-GGTCGTAGGGCTGCTGGAAG-3’

Perfect Plus 1 kb DNA Ladder (EURx) was used as a ladder. 

### 2.7. Extraction of RNA and the Reverse Transcription Reaction 

RNA from the cell lines was extracted using a GeneMATRIX Universal RNA/miRNA Purification Kit (EURx) according to the vendor’s instruction. cDNA was obtained from RNA by performing RT-PCR with random primers (Promega, Madison, WI, USA) and Moloney murine leukemia virus MMLV reverse transcriptase (Promega) according to the manufacturer’s protocol. 

### 2.8. Real-Time PCR

Quantitative real-time PCR gene expression was estimated with the application of TaqMAN probes (Applied Biosystems, Foster City, CA, USA)—human: *GAPDH* (Hs99999905_m1), *MYOD* (Hs00159528_m1), *MEF2A* (Hs01050409_m1), *MYOSTATIN* (Hs00976237_m1), *MYOGENIN* (Hs01032275_m1), *MYH2* (Hs00430042_m1), *HDAC1* (Hs02621185_s1), *HDAC2* (Hs00231032_m1), *SNAI1* (Hs00195591_m1), and Blank qPCR Master Mix (2x) (EURx)—using the Quant Studio 7 Real-Time PCR System (Applied Biosystems). The mRNA expression level for all samples was normalized to the housekeeping gene GAPDH and the 2^−Δ^ or 2^−ΔΔCT^ method allowed us to calculate the relative expression level of the genes. 

### 2.9. Western Blot

The nuclear and cytoplasmic fractions of proteins were isolated with a Nuclear Extract Kit (Active Motif, La Hulpe Belgium) according to manufacturer’s instruction. The protein concentration was measured with a Bradford reagent (BioRad, Hercules, CA, USA) using a Tecan Spark 10 M microplate reader (Tecan Trading AG, Männedorf, Switzerland). The electrophoresis on a sodium dodecyl sulfate-polyacrylamide gel (4% and 12%) and transfer of proteins to polyvinylidene diflouride membrane (BioRad) was performed. The blot was incubated with 1% BSA for 1 h and then overnight with primary antibodies, followed by incubation with secondary antibodies conjugated with horseradish peroxidase and visualized with SuperSignal™ West Pico Chemiluminescent Substrate (Thermo Scientific) using a Gel Doc Imaging System (BioRad). The Western blot was done with anti-SNAI1 mouse mAb (1:1000, L70G2, Cell Signalling) and anti-Histone H3 rabbit pAb (1:1000, ab1791, Abcam, Cambridge UK). Secondary antibodies were conjugated with Horseradish Peroxidase (1:3000, Santa Cruz Biotechnology). 

### 2.10. Sequencing 

Genomic DNA was isolated, as described in the analysis of RH30 clones. It was used for the PCR reaction with AmpliTaq Gold™ 360 DNA Polymerase (Thermo Scientific) with primers overlapping the deletion region, which were the same used with clones screening. Bands with sizes around 500 bp for edited clones and 1200 bp or wild type (WT) were cut from the gel and purified with a GeneMATRIX BASIC DNA Purification Kit (EURx) according to the vendor’s protocol. Concentrations were measured with Nanodrop or Quawell Q5000. To amplify the purified product, the PCR reaction was performed with the same primers and AmpliTaq Gold™ 360 DNA Polymerase (Thermo Scientific). For sequencing, we used a BigDye™ Terminator v3.1 Cycle Sequencing Kit (Thermo Scientific) according to the vendor’s protocol and a 3500 Series Genetic Analyzer (Thermo Scientific).

### 2.11. Visualization of Cellular Morphology

The morphology of the cells was analyzed using an Olympus IX70 microscope (Olympus Corporation, Tokyo, Japan) and a Canon EOS1100D digital photo camera (Canon Inc., Tokyo, Japan).

### 2.12. Statistical Analysis

Unless stated otherwise, the results show the mean ± standard deviation (SD) of at least two to three independent experiments. Statistical analysis was performed using one-way analysis of variance (ANOVA) with Dunnett’s post-test using GraphPad Prism 7.04 software. Differences with a *p*-value of less than 0.05 were considered statistically significant. 

## 3. Results

### 3.1. Generation and Verification of CRISPR/Cas9 and TALEN Systems Targeting SNAI1

**Figure 1:**
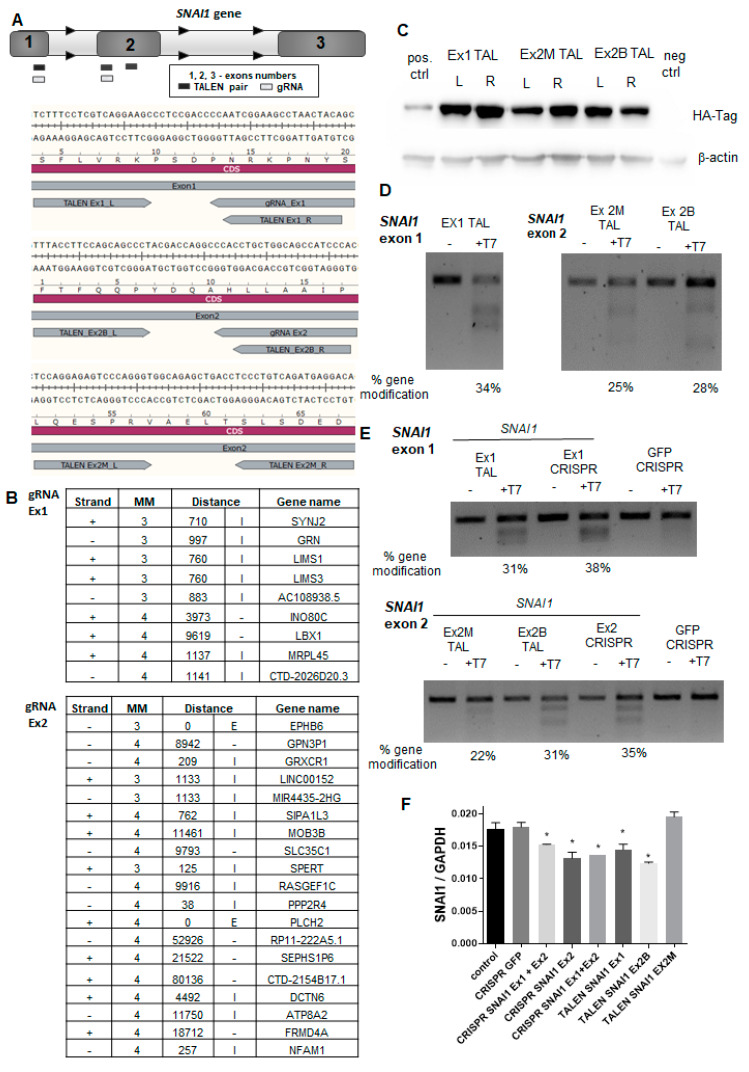
The design and cleavage activity of transcription activator-like effector nucleases (TALEN) and clustered regularly interspaced short palindromic repeats (CRISPR)/Cas9 nucleases targeting the *SNAI1* gene. (**A**) Scheme presenting TALEN pairs and CRISPR/Cas9 nucleases targeting *SNAI1*. (**B**) Analysis of possible off-targets of gRNA1 and gRNA2 was performed with online predictor CCTop (MM—mismatch, E—exon, I—intron). (**C**) Expression of TALENs in transfected HEK293T cells detected with an anti-HA tag antibody using Western blot analysis. (**D**) Cleavage activity of TALENs in transfected HEK293T cells (T7 endonuclease assay). (**E**) Cleavage activity of TALENs and CRISPR/Cas9 nucleases in RH30 cells (T7 endonuclease assay). (**F**) Expression of *SNAI1* mRNA in RH30 cells after transfection calculated as a relative expression of *SNAI1* to *GAPDH* using the ΔCT method (n = 2), * *p* < 0.05. The estimated gene modification in the T7 assay was calculated using the following formula: % gene modification = 100 × (1 – (1 − fraction cleaved)^1/2^).

Our goal was to disrupt the *SNAI1* gene in RH30 cells using concomitant targeting of exons 1 and 2. This induced a deletion that could be easily identified using a PCR of genomic DNA. Therefore, specific TALEN pairs and gRNA sequences recognizing *SNAI1* exon 1 or exon 2 were designed (Figure 1A).

To find the most effective designer nuclease, we performed side-by-side comparisons. To evaluate the specificity of the CRISPR/Cas9 nucleases, CCTop software [37] was employed to identify the most probable off-targets (Figure 1B). All identified potential off-targets contained at least three mismatches. gRNA1 did not overlap with any exon sequence, whereas gRNA2 might have targeted EPHB6 with three MM and PLCH2 with four MM. Other off-targets were in intronic or intergenic DNA (Figure 1B). TALEN specificities were evaluated with PROGNOS software according to the homology and RVD ranking systems [38]. With four mismatches set per half-site of TALEN pair, none of the off-targets were found in exons, promoters, introns, or intergenic regions. Because TALEN were tagged with hemagglutinin (HA), their expression in transfected HEK293T cells was detected with the anti-HA tag antibody (Figure 1C). Next, the cleavage activity of TALEN was evaluated in the transfected HEK293T cells (Figure 1D) and in RH30 cells (Figure 1E) using the T7 endonuclease 1 assay [35]. We also evaluated the expression of *SNAI1* mRNA in RH30 cells after the transfection (Figure 1F). In combination, those analyses enabled us to select the best nucleases for further research and development of RH30 *SNAI1* knockout cells, i.e., TALEN pairs (Ex1 TALEN and Ex2B TALEN) and CRISPR/Cas9 nucleases targeting exons 1 and 2 of the *SNAI1* gene. 

### 3.2. Genome Editing of ARMS with CRISPR/Cas9 and TALEN

**Figure 2:**
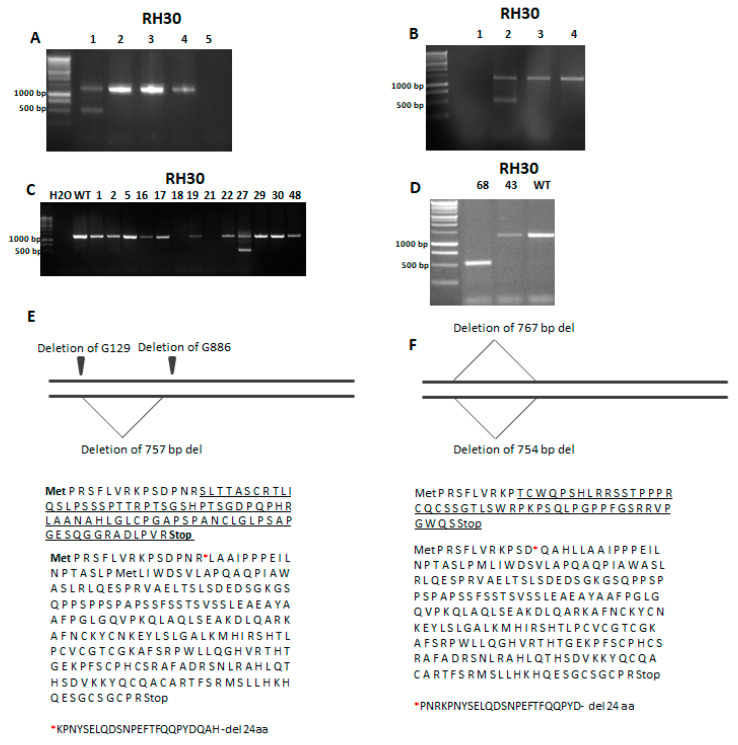
Identification of RH30 clones with a *SNAI1* knockout (KO) after genome editing with TALEN and CRISPR/Cas9 nucleases. (**A**) PCR verification of the activity of CRISPR/Cas9 system (1—RH30 mix after transfection, 2,3,4—RH30 WT, 5—H_2_O). (**B**) Investigation of TALEN activity with PCR using gDNA (1—H_2_O, 2—RH30 mix after transfection, 3—RH30 ctrl, 4—RH30 WT). (**C**) Screening of the clones after CRISPR/Cas9 genome editing using PCR with gDNA. One clone (no. 27) gave two products displaying different sizes. (**D**) Analysis of clones using PCR with gDNA. Clone no. 68 (1) gave only one, shorter product of PCR. (**E**) Sequencing of RH30-27 revealed two point mutations on one allele and one deletion on the other allele. Changes in the protein sequence are underlined. After the 757 bp deletion, the protein sequence was truncated by 24 aa. (**F**) Sequencing of RH30-68 DNA revealed deletions leading to the underlined changes in the protein sequence, the premature STOP codon, and the 24 aa deletion.

To establish the RH30 *SNAI1* KO cell lines, plasmids coding for Cas9 and gRNAs targeting *SNAI1* exons 1 and 2 or TALENs targeting *SNAI1* exons 1 and 2 were introduced to RH30 cells via transfection with Lipofectamine 2000. This strategy enabled us to delete the *SNAI1* gene fragments between two exons. The effectiveness of the editing with CRISPR/Cas9 and TALEN was verified using gDNA from the whole population of cells 48 h after transfection. The PCR reactions with primers targeting exon 1 upstream and exon 2 downstream of the custom nucleases target sites were used to validate the rearrangement of the *SNAI1* gene with the deletion (Figure 2A,B).

If both exons were modified and the repair mechanisms resulted in joining those two breaks, the PCR product should be around 500 bp, whereas the detection of only one band of 1256 bp size indicated that there was no modification or only a few nucleotides were changed on one allele. The detection of two bands indicated a heterozygote with only one allele with the desired deletion. The most favorable was the result with a detection of only one PCR product that was 500 bp in size because it indicated that the editing of both alleles led to the large deletion between exons as an effect of NHEJ (Figure 2A,B). After promising results indicating the efficient DNA rearrangements in both approaches, the isolation of single-cell clones was performed. Screening of the clones revealed that from the 49 clones obtained after CRISPR/Cas9 cleavage, only one (RH30-27) had the deletion on one allele (Figure 2C) and only one clone (RH30-68) from 70 clones obtained after being cleaved with TALEN displayed modifications on both alleles (Figure 2D). Because those two clones displayed deletions of *SNAI1* gene fragments, they were selected for further analysis. Subsequently, Sanger sequencing of *SNAI1* gene was performed to detect point mutations. It revealed that the RH30-27 clone displayed the deletion of a 757 bp fragment on one allele and two point mutations, namely the deletion of G129 and G886 on the second allele (Figure 2E). Analysis of the changed coding sequence (CDS) and a sequence of the new protein revealed that the 757 bp deletion led to the 24 amino acids being absent after R15. Clone RH30-68 obtained after genome editing with TALEN displayed two big deletion fragments of 754 bp and 767 bp in size (Figure 2F). The first one resulted in a 24-amino-acid deletion in the protein sequence after D12, whereas the second deletion led to a frameshift mutation and the presence of a premature STOP codon (Figure 2F).

### 3.3. Analysis of SNAI1 Expression Level

**Figure 3:**
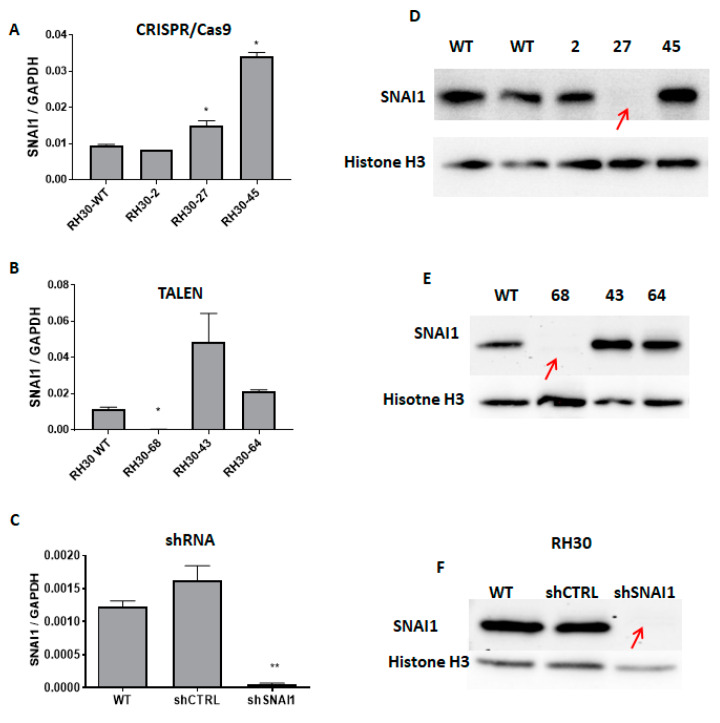
SNAI1 expression levels after modifications with TALEN, CRISPR/Cas9 nucleases, and shRNA. (**A**) *SNAI1* mRNA expression levels in RH30 clones after CRISPR/Cas9 cleavage (qPCR). The data were calculated as a relative expression of *SNAI1* to *GAPDH* using the ΔCT method (n = 2–3). (**B**) *SNAI1* expression levels in RH30 clones after TALEN cleavage (qPCR). A significant reduction of the *SNAI1* expression in clone 68 was observed compared to the WT. The data were calculated as a relative expression of *SNAI1* to *GAPDH* using the ΔCT method (n = 2–3). (**C**) Expression level of *SNAI1* mRNA after transduction with shRNA lentiviral vectors. The data were calculated as a relative expression of *SNAI1* to *GAPDH* using the ΔCT method (n = 3). (**D**) Western blot analysis of the SNAI1 protein expression in clones 2, 27, and 45 after the CRISPR/Cas9 GE. Clone no. 27 showed a complete loss of SNAI1 expression. (**E**) Western blot analysis of SNAI1 protein expression in several clones after TALEN GE. Clone 68 showed the residual expression of the truncated SNAI1 protein. (**F**) Expression of SNAI1 protein in cells after transduction with shRNA lentiviral vectors. * *p* < 0.05. ** *p* <0.01.

Another approach used in our study was based on the stable knockdown (KD) of *SNAI1*, which was obtained as a result of the lentiviral transduction of RH30 cells, as described previously [30]. The lentiviral particles used in our studies were a mixture of three different shRNA sequences targeting *SNAI1*. Control cells (shCTRL) were modified with a vector encoding scrambled shRNA. The stably transduced cells were selected with puromycin, which enabled us to obtain a mix of the cells with the downregulated SNAI1 levels. The clones with a deletion between the two exons, as well as several clones displaying the altered morphology, were characterized to validate the *SNAI1* mRNA expression levels using qPCR. We analyzed the cells modified with CRISPR/Cas9 nucleases (Figure 3A) and TALEN (Figure 3B).

Interestingly, in RH30-27 cells, the *SNAI1* mRNA levels were not diminished (Figure 3A). The Taq-Man probe used in the qPCR recognized the *SNAI1* transcript overlaying the junction of exons 1 and 2. Therefore, it may still have recognized the remaining transcript of the gene after the modifications since the sequencing results revealed point mutations on one of the alleles. The *SNAI1* expression levels were significantly decreased in the RH30-68 clone obtained after TALEN editing in comparison with the WT cells (Figure 3B). Modulation of the SNAI1 expression with shRNA resulted in a significant decrease in the *SNAI1* mRNA levels in comparison to the shCTRL and WT cells. The protein analysis using Western blot showed that all three models (RH30-27, RH30-68, and shSNAI1) displayed significantly diminished expressions of SNAI1 protein (Figure 3D–F) in comparison to the controls. The residual expression of the truncated form of protein could still be observed in the clone RH30-68. In the case of clone RH30-27, the decreased SNAI1 protein expression levels indicated that two point mutations changed the reading frame, resulting in a premature STOP codon. The lack of protein expression suggests that a new form of SNAI1 protein after the 24-amino-acid deletion could not be correctly folded or the antibody could not recognize the epitope any longer.

### 3.4. The Effect of SNAI1 on Rhabdomyosarcoma Development

**Figure 4:**
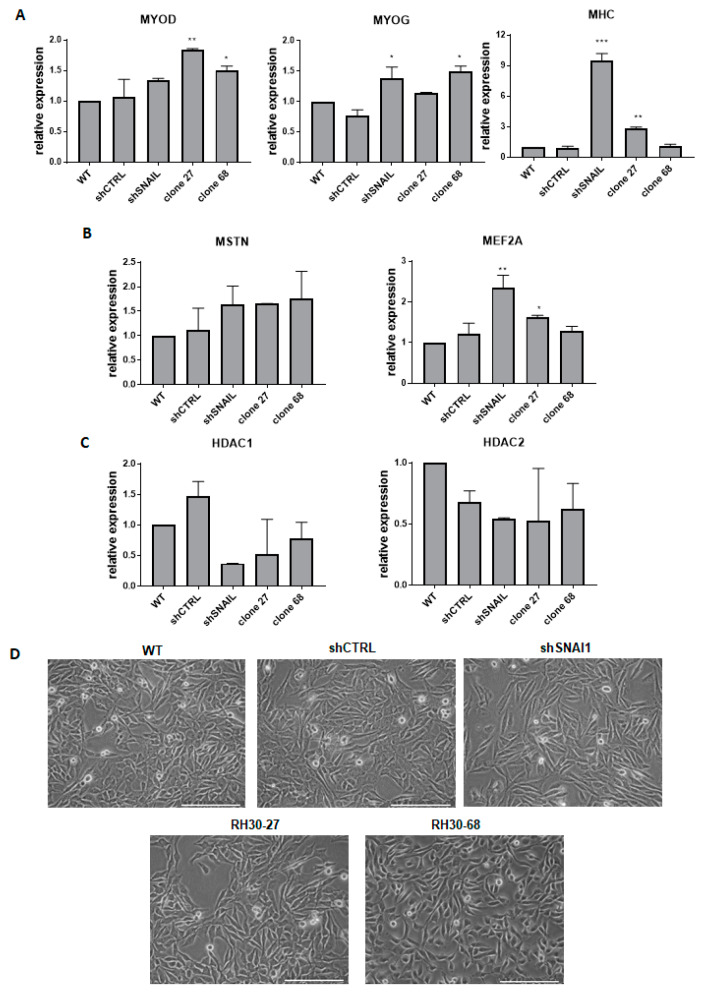
The effect of SNAI1 on rhabdomyosarcoma development. (**A**) qPCR analysis of myogenic regulatory factors: *MYOD, MYOG,* and *MHC* in the *SNAI1* KOs and KDs (n = 2). (**B**) mRNA levels of genes associated with myogenic differentiation *MSTN* and *MEF2A* in the cells after the gene expression modulation (n = 2). (**C**) *HDAC1/2* mRNA levels in the *SNAI1* KOs and KDs (n = 2). (**D**) Morphology of the cells transduced with shRNA lentiviral vectors or modified with TALEN and CRISPR/Cas9 nucleases. The white scale bar represents 200 µm. The mRNA expression levels for all samples were normalized to the housekeeping gene *GAPDH* levels using the 2^−ΔΔCT^ method. * *p* < 0.05, ** *p* < 0.01, *** *p* < 0.001. MYOD: myogenic differentiation 1, MYOG: myogenin, MHC: myosin heavy chain, MSTN: myostatin, MEF2A: myocyte enhancer factor 2A, HDAC1/2: histones deacetylase 1/2.

All of the established models in our study were characterized by the downregulation of SNAI1 levels, but they differed in the transcript levels and incorporated mutations. Because the study aimed to develop the best model for the investigation of the role of SNAI1 in RMS development, we subsequently investigated the effects of *SNAI1* knockout and knockdown on the morphology of the cells and the expression of genes associated with myogenic differentiation. RMS is a tumor that originates from an impaired myogenic differentiation and SNAI1 has been suggested previously as a crucial regulator of myogenic differentiation [30]. To investigate whether different methods of SNAI1 modulation lead to similar biological effects in RMS cells, we first evaluated the expression of the selected regulators of myogenic differentiation in the obtained RH30 *SNAI1* KO and KD cell clones. It was previously shown that decreased SNAI1 expression levels are associated with upregulation in the expression of genes or activity of proteins regulating myogenic differentiation, such as myogenic differentiation 1 (MYOD), myogenin (MYOG), myosin heavy chain (MHC), myocyte enhancer factor 2A (MEF2A), and myostatin (MSTN) [30]. Our results showed a similar pattern of regulation, but at different levels for different approaches. shSNAI1 cells showed significant changes in the levels of late myogenic regulatory factors, such as *MYOG* and *MHC*. From the clones edited using custom nucleases, the RH30-27 clone displayed elevated levels of *MYOD* and *MHC*, whereas the RH30-68 clone displayed increased levels of *MYOD* and *MYOG* (Figure 4A).

*MSTN* was regulated at a similar level in all three cases, but the shSNAI1 cells displayed the strongest effects on the *MEF2A* level (Figure 4B). Other important gene candidates associated with SNAI1 activity are histones deacetylases HDAC1 and HDAC2 that have been shown to interact with SNAI1 in the regulation of E-cadherin expression [39]. Furthermore, in differentiating RMS cells, SNAI1 forms a repressive complex with HDAC1/2 and regulates their expression [30]. Our results unveiled a tendency toward diminished expression levels of both *HDAC1* and *HDAC2* after SNAI1 downregulation in KO and KD cells (Figure 4C). Acquisition of the spindle-shaped morphology of the RMS cells has been previously associated with a myogenic differentiation of cells [30]. Therefore, the morphology of the cells was also investigated in our models. Interestingly, cells with downregulated SNAI1 levels changed their morphology into more elongated, spindle-shape-like cells compared to the shCTRL cells or WT cells. A similar tendency was visible in RH30-27 and RH30-68 clones (Figure 4D). Nevertheless, the most pronounced changes in the morphology of the cells were observed for cells transduced with shRNA vectors.

To conclude, we have established three different models for the investigation of the role of SNAI1 in RMS development. All three models displayed significant changes in the expression levels of genes associated with myogenic differentiation and cellular morphology, and all three models can be utilized in the future to investigate the mechanism of SNAI1 action in RMS differentiation, growth, and metastasis.

## 4. Discussion

In the current study, three different methods of gene expression modulation were compared. A multiplex genome editing approach was performed to establish *SNAI1* KOs in RMS cell lines with TALEN and CRISPR/Cas9 nucleases, whereas *SNAI1* KDs were generated with shRNA. 

Gene silencing with shRNA only allows for knockdown and some residual expression is usually present. For genes crucial for cell survival, this approach might be the only one that can reveal their function. The shRNA gene regulation mechanism depends on the complementarity level with the target sequence, results in a protein KD, and could led to a transcript degradation. After the gene expression modulation with shRNA technology, one can work on the whole cell population. When delivery of the system is done using the lentiviral vectors, the desired cells are usually obtained via selection with dedicated antibiotics or via sorting [30]. Nevertheless, clonal selection is also possible, but it was not used in our studies. Plasmid application efficiency is currently limited by the efficiency of transfection/electroporation. With shRNA technology, a control cell line modified with a scrambled shRNA vector is required to exclude the insertional mutagenesis effect [6]. 

The mechanism underlying the *SNAI1* KO with genome editing (GE) systems is based on modification of the DNA sequence. Therefore, both mRNA and protein levels could be affected. Depending on the type of modification ensuing after the GE, expression changes might, but must not, appear at the transcript level [40]. As was shown in our study, mRNA downregulation was observed in the TALEN-edited cell clone, whereas the *SNAI1* transcript level was not affected in the CRISPR/Cas9-edited clone. The Taq-Man probe used in the qPCR analysis recognized a *SNAI1* exon junction, and was hence able to detect transcript levels if no significant changes in the junction sequence were present. Analysis of both the mRNA and protein expression levels is necessary to correctly analyze the results, which required confirmation in the sequencing of the edited fragment. The sequence analyses of RH30-27 and RH30-68 clones confirmed the presence of premature STOP codons. The expected truncated forms of the protein could not be detected using Western blotting. This might be the effect of incorrect protein folding, which may have resulted from the degradation or the modified protein could not be recognized by the antibody [41]. The vendor of the monoclonal antibody declares that it is produced by immunizing animals with a recombinant human SNAI1 protein. 

The application of genome editing technology always requires clonal selection because in each cell, different modifications may take place. Therefore, different effects might be obtained. Several clones should be examined to exclude the effect of off-targets and to confirm the specificity of the observed biological effect. Unfortunately, the overall efficiency of the editing might be low because of many factors, such as the effectivity of the introducing system to the cells, the repair mechanisms, and the types of gRNA, where all of them may exert an effect [41,42]. Moreover, only editing one of the alleles is another hurdle [43]. This leads to the necessity of the screening of many clones. We analyzed 49 CRISPR/Cas9 clones and 70 TALEN clones, which resulted in only one clone being positive for deletion between the exons in each approach. This is a very low efficiency, especially taking into consideration the initial analysis of the mixed cell population after transfection indicated quite promising results. The viability of RH30 clones may have been affected by a *SNAI1* KO. As it was shown in our previous paper, an RH30 *SNAI1* KO cell population tends to be diminished during the cell culture. 

The application of reporter and selective markers in this approach represents a possibility to work on a cell population after CRISPR/Cas9 genome editing [30]. Currently, many selective or reporter genes are available with the CRISPR/Cas9 system, such as antibiotics resistance gene or fluorescence proteins, which encourages the use of this tool. The modification of Cas9 or *FokI* opens a variety of new applications for those systems, such as transcription activation, epigenetic modification, visualization, or purification of particular genomic fragment [44,45]. Depending on the delivery technique, insertional mutagenesis may challenge the application of those systems. Recently, the introduction of the CRISPR/Cas9 system to the cells as a ribonucleoprotein complex was widely shown to diminish off-targets effects [46].

One of the main hurdles in the application of gene expression regulation systems is the presence of off-targets. They may occur as an effect of mismatch between the target sequence and the element responsible for the specificity of a particular technology: shRNA, gRNA, or TALEN. It was not possible to validate the off-target effects for the shRNA targeting *SNAI1* because the producer does not provide their sequences. Nevertheless, in our previous studies, we validated the specificity of that model via the restoration of the SNAI1 level. RH30 shSNAI1 cells were transduced with viral vectors encoding *SNAI1*. Restoration of the SNAI1 level in RH30 shSNAI1 reversed the effects of SNAI1 silencing on HDAC1/2 levels and tumor growth in NOD-SCID mice [30]. To evaluate the precision of the gRNA used in this study, we identified and ranked all gRNAs target sites according to their off-target quality. We found a total of 28 off-target sites (9 for Ex1 gRNA and 19 for Ex2 gRNA) that could be potentially targeted using our CRISPR-Cas9 nuclease. However, only two of these sites were located within exonic regions of the genes, namely in *EPHB6* (tyrosine-protein kinase-defective receptor) and *PLCH2* (phospholipase C eta 2). The obtained results did not identify any potential off-target site in the proximity of genes that are directly associated with myogenic differentiation. However, some of them might be important for the cell shape or cancer development, such as *EPHB6* in tumor invasion and metastasis [47], *LIMS1* (LIM zinc finger domain containing 1) in cell survival and differentiation [48], *SIPA1L3* (signal-induced proliferation-associated 1-like protein 3) in cell polarity and cytoskeleton organization [49], or *PPP2R4* (protein phosphatase 2 phosphatase activator) in cell growth and division [50]. Therefore, in the future, experimental validation of the cleavage at the potential off-target sites identified *in silico* should be performed in the *SNAI1* KO cells. To verify the off-target effect caused by the unspecific activity of TALEN, we performed analyses based on the homology and RVD ranking with four mismatches given [51] using the same strategy as for CRISPR/Cas9. No off-target activity was reported with this setting, which implies a high specificity of generated nucleases (four mismatches). Nevertheless, the first off-target was reported when the TALEN Ex1 pair had three and five mismatches in the left and right half-sites, respectively. In TALEN Ex2 B, two possible off-targets occurred after two and six mismatches in the left and right half-sites, respectively. First, the unspecific TALEN Ex2 M activity might have been possible with one mismatch in the left half-site and six mismatches in the right half-site of the pair. Nevertheless, we believe that the use of three different methods to modulate the SNAI1 levels led to similar biological results, therefore limiting the contribution of off-target effects to the observed phenotype. Working on a small number of clones instead of a whole population might be the reason for the slight discrepancy in the expression levels of myogenic differentiation markers and other genes. To diminish the likelihood that off-target cleavage might have occurred, many approaches could be applied. In the case of CRISPR/Cas9 nucleases, the use of nickase via inactivation of one of the catalytic domains of the Cas9 might be implemented even though it typically results in an overall decreased cleavage activity [44]. The customized concentration of RNAi or GE tools, as well as the temporal expression of custom nucleases, negatively correlates with unspecific activity. Additionally, the application of non-targeting gRNA control, the confirmation of on target KO/KD effect with rescue experiment, and the employment of different approaches may also be used [52]. After a CRISPR/Cas9 application, huge rearrangements may be observed in primary and cancer cell lines [53]. Whole-genome sequencing is rarely used in this kind of study, but its potential should not be omitted. 

As was shown in our previous study, SNAI1 is a crucial factor that regulates the myogenic differentiation in ARMS cells [30,54]. Since RMS is a tumor originating from the impaired myogenic differentiation of stem cells, it will be possible in the future to develop new treatment strategies based on the induction of myogenic differentiation of tumor cells to inhibit its growth [55]. To verify whether our KOs and KDs exerted an effect on the myogenic differentiation of RMS, an analysis of the myogenic regulatory genes expression levels was performed. The obtained data show different levels of expression between the clones and shSNAI1, especially in the case of *MHC*. However, the main trend in the expression was similar in SNAI1 KOs and KDs. In shSNAI1, the increased expression of *MYOG*, *MHC*, and *MEF2A* was observed; in RH30-27, *MYOD*, *MHC*, and *MEF2A* was observed; and in RH30-68, *MYOD* and *MYOG* was observed. All of the KOs and KDs showed a slight upregulation of *MSTN* and downregulation of *HDAC1* and *HDAC2*. The slightly different levels of gene expression might be related to the residual expression of the truncated form of SNAI1 in RH30-68 or the presence of the *SNAI1* transcript in RH30-27. In future, the phenomenon of SNAI1′s role in myogenic differentiation in the new RMS models requires further study.

## 5. Conclusions 

To conclude, in this work, we used three different approaches to obtain the best model for further study on RMS development. We compared those methods using the analysis of DNA, mRNA, protein, and off-target discovery. Despite the different mechanisms of gene silencing, the described methods led the same biological effect, namely they resulted in the deregulation of genes associated with myogenic differentiation, which is in accordance with the elongated, spindle-like morphology of the cells, which was the most visible form in the shSNAI1 cells. The establishment of a reliable model constitutes a basis for further experiments on the precise mechanism of SNAI1 action. Furthermore, those methods could be applied to obtain knockouts in different types of cell lines, including stem cells.
